# Analysis of Solubility, pH, Antimicrobial Action and Cytotoxicity of Calcium Hydroxide Paste Associated With Ambroxol Hydrochloride

**DOI:** 10.1111/aej.70039

**Published:** 2025-11-22

**Authors:** Thaís Ferreira Rodrigues Mota, Ana Flávia Balestrero Cassiano, Pedro César Gomes Titato, Pedro Henrique Souza Calefi, Murilo Priori Alcalde, Gisele Faria, Marco Antonio Hungaro Duarte

**Affiliations:** ^1^ Department of Dentistry, Endodontics and Dental Materials, Faculty of Dentistry of Bauru University of São Paulo Bauru São Paulo Brazil; ^2^ Department of Restorative Dentistry, Araraquara School of Dentistry São Paulo State University – UNESP Araraquara São Paulo Brazil

**Keywords:** ambroxol hydrochloride, antimicrobial action, calcium hydroxide, cytotoxicity, pH

## Abstract

This study evaluated the association of ambroxol hydrochloride with calcium hydroxide paste as intracanal medication to enhance antimicrobial action on biofilm without affecting pH, solubility and cytotoxicity. Calcium hydroxide (CH) paste and formulations with different ambroxol concentrations were placed into artificial teeth conditioned in distilled water. pH and solubility were measured. Dentine discs infected with *E. faecalis* were covered with pastes, and biofilm viability was analysed using live/dead assay and confocal microscope. Cytotoxicity was analysed using MTT assay on fibroblast and osteoblast‐like cells. Data were statistically compared (*p* < 0.05). Ambroxol showed an acidic pH after 7 days, compared to CH, but the 30% concentration became more alkaline after 30 days. While CH's pH decreased over time, ambroxol groups maintained stability. Solubility decreased for all groups over time. All groups showed significant antimicrobial differences from the control group. The association did not alter CH's cytotoxicity. Further research is needed for optimal ambroxol concentration.

## 1 Introduction

Calcium hydroxide has been the most widely used intracanal medication since its introduction by Hermann in 1920 [1]. It is indicated for necropulpectomy and biopulpectomy when there is aseptic chain breakage [2]. Its physicochemical properties, such as a strong base with a high pH (around 12.6) and low water solubility, contribute to its broad‐spectrum antimicrobial action [1, 3, 4]. Its mechanism of action involves protein denaturation in the cytoplasmic membrane via dissociated hydroxyl ions [3]. However, calcium hydroxide's limitations include incomplete removal, which may hinder the adhesion of certain endodontic sealers, and it requires complete contact with tissue, necessitating the canal to be filled homogeneously up to the working length [2]. Furthermore, its effectiveness against *Enterococcus faecalis* biofilms remains limited [2, 3, 5].

*Enterococcus faecalis* is a facultative anaerobic bacterium commonly found in persistent infections. It can resist the main action mechanism of calcium hydroxide through a proton pump present in its cytoplasmic membrane, which prevents pH changes, even resisting values up to pH 11 [1, 6, 7]. Several combinations are being studied to enhance the effectiveness of calcium hydroxide paste, though results are mixed. Some studies report improved antimicrobial action when calcium hydroxide is combined with chlorhexidine [8, 9, 10], while others do not [11]. A study combining calcium hydroxide with chlorhexidine gel and *Casearia sylvestris Sw*. extract found no significant differences [12]. Additionally, studies involving paramonochlorophenol, did not improve antimicrobial action, maintaining high percentages of live microorganisms [10, 13, 14].

Ambroxol hydrochloride (C_13_H_18_Br_2_N_2_O) is a widely used anti‐inflammatory medication, primarily for respiratory and throat diseases in pulmonary medicine, with mucolytic effects [15]. It acts in chemotaxis, serves as a local anaesthetic, and is a potent chemoattractant responsible for initiating the inflammatory response of monocytes and neutrophils after stimulation [11, 15, 16]. It promotes the release of serous mucus in the respiratory system [17], which could be a potential alternative for treating intracanal biofilm. This mucolytic action could provide more effectiveness in the polysaccharide layer of endodontic biofilm. Previous studies have suggested that ambroxol has significant antimicrobial potential against *Enterococcus faecalis* biofilms, although it exhibited higher cytotoxicity than calcium hydroxide [18]. As an intracanal medication, ambroxol is still underexplored, and research on its association with calcium hydroxide paste is scarce.

Thus, this study aimed to evaluate the combination of ambroxol hydrochloride and calcium hydroxide paste to improve antimicrobial activity on biofilm without affecting pH, solubility or cytotoxicity. The null hypothesis was that the addition of ambroxol would not significantly affect pH, solubility or cytotoxicity but would improve antimicrobial action.

## 2 Methodology

### 2.1 pH and Solubility Analysis

The sample calculation to pH and solubility analysis was performed using G*Power v9 for Mac (Heinrich Heine, Universität Düsseldorf) by selecting the ANOVA test of the F test family. The pilot study's data and the present study's effect size were established (= 1.10). The alpha type error of 0.05, a beta power of 0.80 and a ratio N2/N1 of 1 were also stipulated. A total of 08 samples per group were indicated as the ideal size required for noting significant differences. A total of 10 samples per group were used.

Forty artificial upper incisors with standardised canals and foraminal openings of 400 μm (IM do Brasil, São Paulo, SP, Brazil) were divided into four groups (*n* = 10), as follows:

- G1: 100% calcium hydroxide paste (CH);
- G2: 70% calcium hydroxide paste with 30% ambroxol hydrochloride (CH30AB);
- G3: 50% calcium hydroxide paste with 50% ambroxol hydrochloride (CH50AB);
- G4: 30% calcium hydroxide paste with 70% ambroxol hydrochloride (CH70AB).

Propylene glycol was used as the vehicle. The ratio was 1 g of powder per 1 mL of liquid. The teeth were filled using a Lentulo spiral, and their crowns were sealed. The teeth were immersed in 15 mL plastic vials hermetically sealed, each containing 10 mL of deionised water.

#### 2.1.1 pH Analysis

The pH changes were evaluated at 7, 15 and 30 days after paste placement. Each specimen was removed from the flask and immersed in a new tube with the same volume of deionised water for each measurement. The liquid in each group flask was measured using a pH meter electrode, calibrated with known buffers (pH 4.7 and 14). The control group was deionised water, and its pH was measured at all time points.

#### 2.1.2 Solubility Analysis

After filling the pastes, the teeth were weighed individually. The apices were immersed in 10 mL of distilled water. After the 7‐, 15‐ and 30‐day periods, the teeth were reweighed, and the apices were immersed in a new bottle containing 10 mL of distilled water. The solubility percentage was calculated by comparing the initial and final weights.

### 2.2 Antimicrobial and Cytotoxicity Tests

The sample calculation for antimicrobial and cytotoxicity was performed with the data from the pilot study. The pilot study's data and the present study's effect size were established (= 1.10). The alpha type error of 0.05, a beta power of 0.80 and a ratio N2/N1 of 1 were also stipulated. A total of 04 samples per group were indicated as the ideal size required for noting significant differences. A total of 10 samples per group were used.

#### 2.2.1 Antimicrobial Capacity on *Enterococcus faecalis* Biofilm

Dentine discs were obtained from the cervical and middle thirds of bovine incisors using a 4 mm trephine (4 discs per tooth). A polishing machine (Fortel Indústria e Comércio Ltda., SP, Brazil) smoothed and polished the discs, standardised to 2 mm thick. They were sterilised and treated with 1% sodium hypochlorite (Rioquímica Ltda., São José do Rio Preto, SP, Brazil) for 15 min, followed by 17% EDTA (Biodenâmica, Ibiporã, PR, Brazil) for 3 min. The discs were placed in test tubes with lids containing 5 mL of distilled water and sterilised in an autoclave at 121°C.

##### 2.2.1.1 Biofilm Growth

All microbiological procedures were conducted under aseptic conditions in a laminar flow chamber (VecoFlow Ltda, Campinas, SP, Brazil). To activate *Enterococcus faecalis*, 15 μL of the standard strain (29212) was placed in 3 mL of sterile culture medium (BHI) at 37°C for overnight growth. The bacterial density was adjusted to a 1 × 10^9^ concentration and used to inoculate dentine discs. The bacterial density was adjusted by a spectrophotometer (UV‐VISIBLI, Shimadzy, Japan) at an optical density of 1 at 600 nm according to the 0.5 MacFarland standard.

##### 2.2.1.2 Dentine Discs Contamination

The dentine discs were placed in wells of a 24‐well plate, each containing 100 μL of *E. faecalis* and 1900 μL of BHI. The plate was incubated in an oven at 37°C for 14 days [19], with the BHI being replaced every 2 days (2000 μL of BHI at each exchange).

##### 2.2.1.3 Antimicrobial Test

The pastes were applied in the same proportion as previous tests. After the incubation period and maturation of the biofilm, the discs were washed with 1 mL of distilled water to remove planktonic bacteria. The contaminated discs were completely covered with pastes for the direct contact test. All groups including the control group were separated into Petri dishes (*n* = 5). They were then placed in an oven at 37°C for 7 days, the minimum contact period of intracanal calcium hydroxide medication, explored in previous work [13, 14].

##### 2.2.1.4 Microbiological Analysis

After 7 days, the discs were washed out of any culture medium with 1000 μL PBS to remove the pastes and planktonic cells. Biofilm viability was assessed using the SYTO 8/propidium iodide staining technique (Live/Dead BacLight Viability Kit; molecular probes, Eugene, OR). The discs were kept in a dark environment with 15 μL of dye for 15 min. Then, they were directly observed through a confocal laser scanning microscope (Leica TCS‐SPE; Leica Bio‐systems CMS, Mannheim, Germany). Four stacks of random areas were obtained for each sample through a 400× magnification lens. Five samples were obtained per group, 20 ‘stacks’ for each medication. The bioImage_L software (www.bioImageL.com) calculated the total biovolume and the percentage of dead cells found after treatment.

#### 2.2.2 Cytotoxicity

##### 2.2.2.1 Cell Culture

L929 mouse fibroblasts and human osteoblast‐like cells (Saos‐2) were used. The cells were maintained in culture bottles with modified Dulbecco's medium (DMEM—Sigma‐Aldrich, St. Louis, Missouri, USA) containing 10% fetal bovine serum, 100 μg/mL of penicillin and 100 μg/mL of streptomycin under standard cell culture conditions (oven at 37°C, humidified atmosphere containing 5% CO_2_ and 95% air).

##### 2.2.2.2 Paste Preparation, Dilutions and Cell Treatment

The experimental groups were the same as the other tests. Untreated cells were the control group. The pastes were exposed to ultraviolet light for 10 min. After preparation, an aliquot of 100 mg from each group was placed in 1.5 mL microtubes, and 1.2 mL of DMEM medium was added, remaining in an incubator at 37°C for 24 h. The supernatant was transferred to new microtubes and centrifuged for 10 min at 20.800 ***g*** (5430; Eppendorf AG) to decant material particles in the supernatant [20]. Again, the supernatant was transferred to new microtubes, which are considered the ‘mother solution’ (1:1). Then, it was diluted (1:2, 1:4, 1:8, 1:16 and 1:32) and placed in contact with cell lines to obtain a dose–response curve [20, 21].

##### 2.2.2.3 Evaluation of Cell Viability/Metabolism by MTT Assay

Cells were plated in 96‐well culture plates (5 × 10^4^ cells/mL). After 24 h, they were incubated with the medications and controls for 24 h, 48 h and 7 days. After these periods, the supernatant was removed and 100 μL of MTT solution (Sigma‐Aldrich) at 0.5 mg/mL was added. They were incubated for 4 h in an oven with 5% CO_2_ and 95% humidity at 37°C. Then, 100 μL of acidified isopropyl alcohol (HCl: 0.04 N) was added to the extract to solubilise the formazan crystals. The optical densities of the solutions were measured in a spectrophotometer with a 570‐wavelength filter (Asys‐UVM 340, Biochrom‐Mikro Win 2000). The percentage of cell viability was calculated by comparing the absorbance of treated cells, with the negative control, considered to have 100% cell viability.

### 2.3 Statistical Analysis

Data normality was analysed using the Shapiro‐Wilk test. pH and volumetric solubility did not show normal distribution, but with variance < 30%. Cytotoxicity and antimicrobial data showed normal distribution. Statistical analysis was performed using GraphPad Prism 9.0 (GraphPad Software Inc., San Diego, California, USA), with two‐way ANOVA and Tukey's post‐test. The significance level was set at 5%. Post hoc tests are used to locate group differences.

## 3 Results

### 3.1 pH E Solubility

The results are shown in Table 1. The ultrapure water pH was the pH meter's control measure, 6.69.

**TABLE 1:** Mean and standard deviation of pH and percentage of solubility of the pastes in the different periods studied.

	7 days		15 days		30 days	
	pH	% solubility	pH	% solubility	pH	% solubility
Water	6.69 ± 0.06^a,A^		6.69 ± 0.06^a,A^		6.69 ± 0.06^a,A^	
CH	9.37 ± 1.78^b,A^	11.6 ± 2.7^a,A^	7.15 ± 0,2^a,B^	9.2 ± 0.9^a,B^	6.9 ± 0.3^a,C^	7.9 ± 2.2^a,C^
CH30AB	7.69 ± 1.43^a,AB^	12.6 ± 1.1^b,A^	7.18 ± 0.2^a,A^	10.0 ± 1.1^a,B^	7.95 ± 0.95^b,B^	8.6 ± 1.3^a,C^
CH50AB	6.58 ± 0.3^a,A^	7.6 ± 3.5^a,A^	6.96 ± 0.22^a,B^	5.8 ± 2.8^a,B^	6.91 ± 0.17^a,B^	4.2 ± 2.8^a,C^
CH70AB	6.98 ± 0.43^a,A^	6.1 ± 5.4^a,A^	6.25 ± 0.35^b,B^	9.8 ± 10.1^a,B^	6.93 ± 0.18^a,B^	4.3 ± 1.3^a,C^

*Note:* Different lowercase letters indicate statistically significant differences (*p* < 0.05) between the groups. Different capital letters indicate statistically significant differences (*p* < 0.05) between time intervals.

At 7 days, the CH paste presented pH 9.37, differing significantly from other pastes (*p* < 0.05). After 15 days, the CH paste and CH30AB presented pH values statistically significantly different from the CH70AB paste (*p* < 0.05). After 30 days, the CH30AB presented pH 7.95, significantly different values than the other pastes (*p* < 0.05).

The association decreased the paste pH when compared to pure calcium hydroxide paste in 7 days. But in 15 and 30 days, only CH70AB and CH30AB differed from the others. In 15 days, CH70AB showed an acidic pH and in 30 days CH30AB showed an alkaline pH. Over time, CH's pH decreased significantly. CH30AB showed more acidic pH in 15 days and alkaline pH in 30 days, but different from 7 days. CH50AB showed more alkaline pH in 15 and 30 days and CH70AB more acidic pH in those periods.

Concerning solubility, each percentage was calculated by comparing the initial and final teeth weight. Significant differences occurred in 7 days, for CH and CH30AB (11.6 ± 2.7 and 12.6 ± 1.1, respectively), as well as in 30 days (7.9 ± 2.2 and 8.6 ± 1.3, respectively).

### 3.2 Antimicrobial Action

**FIGURE 1:**
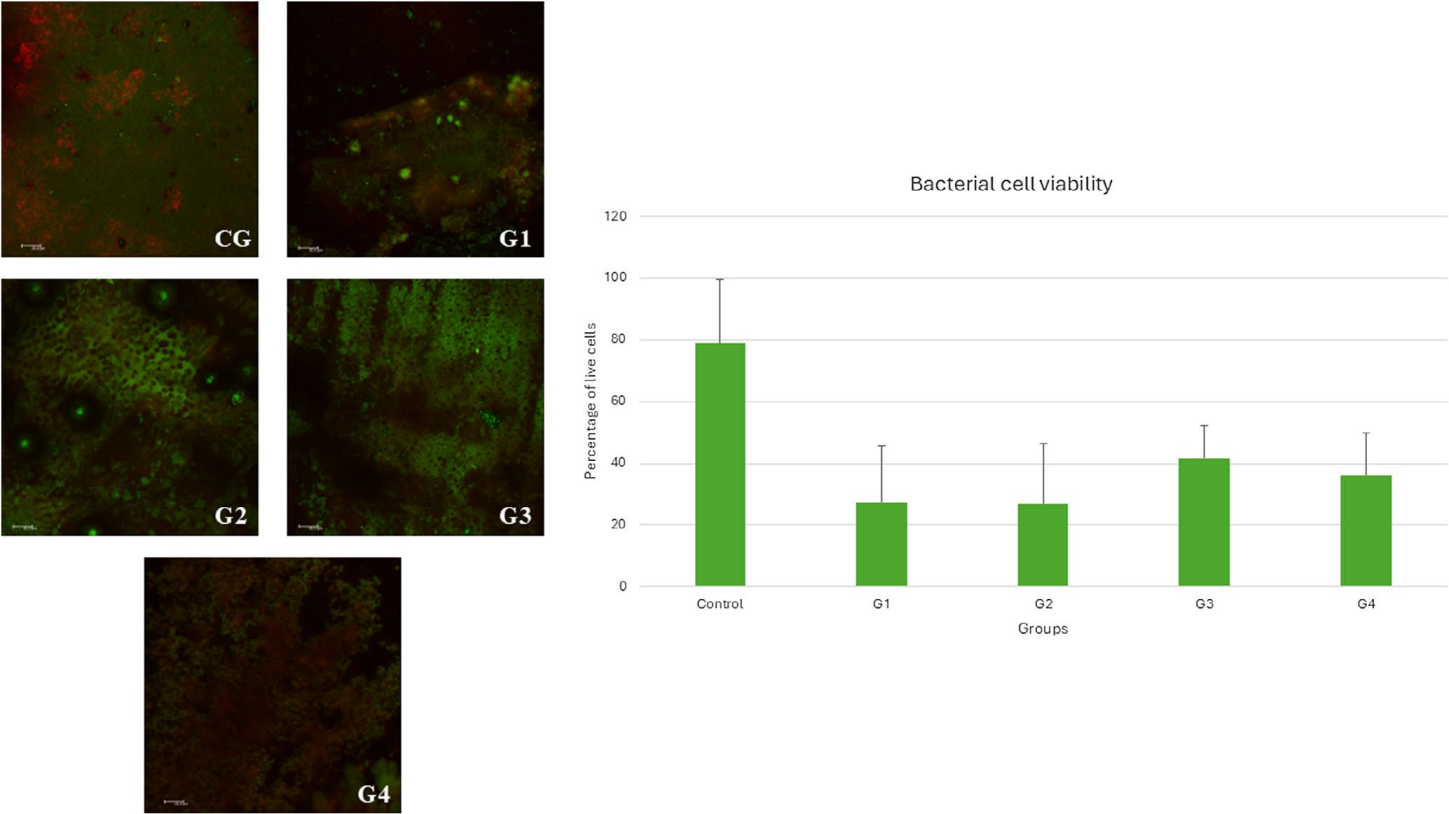
Representative images and % of live and dead of *Enterococous faecalis* after 7 days of contact with the studied paste (G1: 1 g of calcium hydroxide paste; G2: 0.7 g of calcium hydroxide paste with 0.3 g of ambroxol hydrochloride; G3: 0.5 g of calcium hydroxide paste with 0.5 g of ambroxol hydrochloride; G4: 0.3 g of calcium hydroxide paste with 0.7 g of ambroxol hydrochloride).

All treated groups exhibited statistically significant differences from the control group, but no statistical differences were observed among the treatment groups. Figure 1 presents the graphic with the percentage of live and dead cells in the biofilm of *Enterococcus faecalis* after the contact of the cells for 7 days and the representative images.

### 3.3 Cytotoxicity

#### 3.3.1 L929 Cells

**FIGURE 2:**
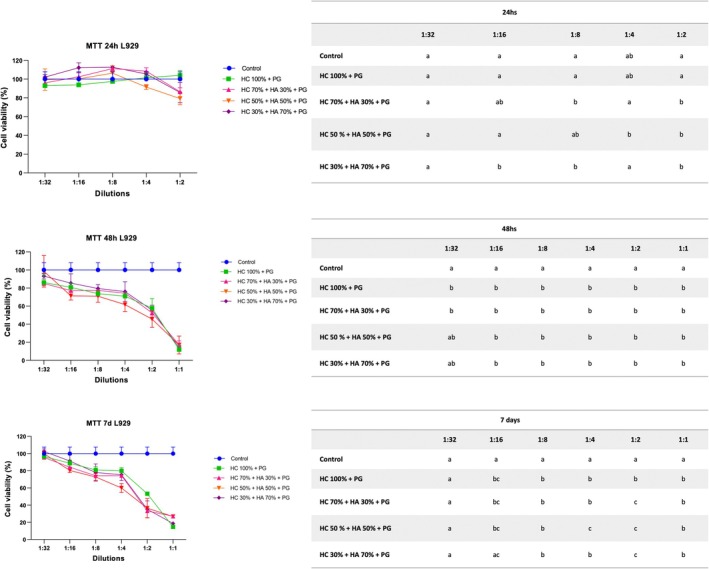
Viability of L929 cells after exposure for 24 h, 48 h and 7 days to different dilutions of the pastes studied using the MTT assay. Statistical comparison of results. Different letters in the lines indicate a statistical difference between the pastes in each dilution.

At 24 h, 100% CH showed no significant difference from the control group—untreated cells (*p* > 0.05). The viability of the cells exposed to the other pastes evaluated, in general, was not statistically different from the control group, except for the dilution of 1:2 (*p* < 0.05). At 48 h and 7 days, all pastes exhibited higher cytotoxicity compared to the control group (*p* < 0.05), and there was no significant difference between them (*p* < 0.05) (Figure 2).

#### 3.3.2 SAOS Cells

**FIGURE 3:**
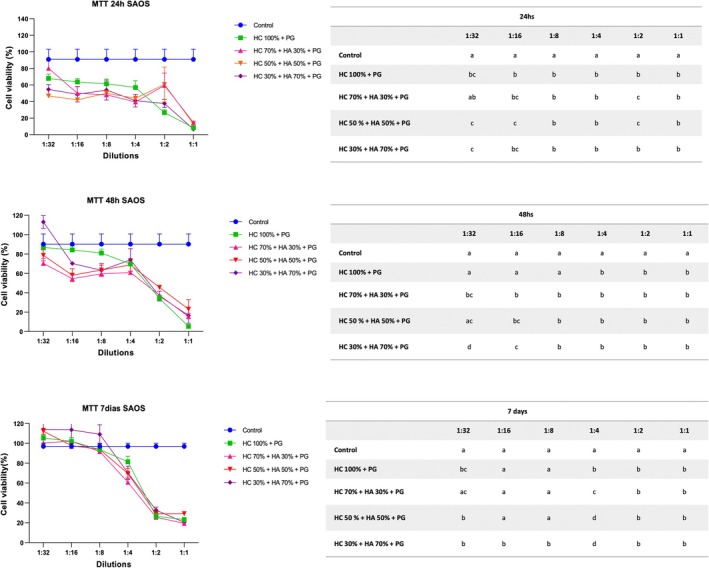
Viability of SAOS cells after exposure for 24 h, 48 h and 7 days to different dilutions of the pastes studied using the MTT assay. Statistical comparison of results. Different letters in the lines indicate statistical differences between the pastes in each dilution.

At 24 h, all pastes were more cytotoxic than the control group (*p* < 0.05). In general, at 48 h, all pastes showed similar results in the dilutions evaluated (*p* > 0.05). In the 7‐day period, all the pastes evaluated were more cytotoxic than the control group when more concentrated (1:4, 1:2 and 1:1) (*p* < 0.05). However, there was no statistically significant difference among themselves in the dilutions evaluated (Figure 3).

## 4 Discussion

The high pH property was verified by measuring the pH of distilled water that previously contained different groups of teeth. Groups with higher amounts of calcium hydroxide paste (G1 and G2) demonstrated the most basic pH values [1, 3, 4]. This may be related to the acidic pH of ambroxol hydrochloride [18], in addition to the smaller foraminal opening, which provides less output of hydroxyl ions, thereby reducing the pH of calcium hydroxide itself [18]. The association between these factors may have influenced the pH, as ambroxol hydrochloride is more soluble in water than calcium hydroxide. The H^+^ ions released by the salt are sufficient to neutralise all the OH^−^ released by the base, resulting in an acidic environment, as hydroxonium remains in solution.

Regarding solubility, all groups showed a decrease in their percentage over time. After 7 days, CH30AB showed higher solubilisation compared to other groups. However, after 15 and 30 days, no significant differences were observed between the groups. This could be due to the pH's change. pH interferes with solubility. This new formulation resulting from the union of the pastes may have helped in the formation of larger particles. As larger particles tend to reduce dissolution efficiency [22, 23]. Another possibility is the use of propylene glycol as a vehicle, which has shown higher solubilisation than other vehicles in previous studies [13].

Concerning the antimicrobial action against *E. faecalis* , this study sought a satisfactory association between the two intracanal medications. The antimicrobial action of calcium hydroxide was not impaired and could reduce the microbial load. Godoy [24] demonstrated bacterial organisation in biofilm, colonies and planktonic bacteria, which were present in abundance in deciduous teeth with pulp necrosis. Biofilm formation contributes to bacterial resistance to medications. Previous studies have shown ambroxol's ability to inhibit biofilm formation in multidrug‐resistant bacteria, suggesting that its mechanism of action targets dehydrosqualene synthase. This molecule contributes to the synthesis of the golden carotenoid pigment staphyloxanthin, an antioxidant that helps bacteria survive inside cells [25]. None of the groups showed significant differences, but all demonstrated antimicrobial effects compared to the control group.

In comparison with previous studies, ambroxol itself showed an acidic pH and higher solubility percentage than calcium hydroxide, as well as better antimicrobial action [18]. However, this data was not reported in this study, possibly due to the association between two medications. The interaction between these materials might affect the ideal pH and solubility values of each, reducing their overall effectiveness. This may also demonstrate the efficiency of ambroxol hydrochloride in targeting intracanal biofilm, as reported in previous studies [18], without interfering with the antimicrobial action of calcium hydroxide.

Endodontic materials may come into direct contact with cells from the periapical region, such as fibroblasts, osteoblasts and undifferentiated mesenchymal cells. Therefore, fibroblast and osteoblast‐like cells were used in this study. L929 and Saos‐2, immortalised cell lines, are commonly used to evaluate the cytotoxicity of endodontic materials [26, 27], and are recommended by ISO standards for testing new dental materials [27]. The MTT assay was used to assess mitochondrial succinate dehydrogenase activity. This enzyme converts yellow tetrazolium salt into coloured formazan compounds, with absorbance proportional to the number of metabolically active/viable cells (ISO 10993‐5, 2009). In some cases, the cell culture model does not allow the application of undiluted intracanal medicament extracts to cells, as high concentrations can cause total cell death, preventing the comparison of medicament cytotoxicity. To overcome this limitation, extracts were diluted at various concentrations (up to 1:24) to establish a dose–response curve [20, 21]. From a clinical perspective, this curve is also relevant because certain substances induce more pronounced cellular alterations at lower concentrations [28]. Additionally, this approach is important because, once the material comes into contact with tissues, it releases leachable compounds that are gradually cleared by extracellular fluids, leading to a progressive reduction in their concentration [29]. To simulate these in vivo conditions over time, different dilutions of intracanal medicaments were tested [20, 21]. The results showed that ambroxol hydrochloride did not affect the biological compatibility of calcium hydroxide at these concentrations. This contrasts with previous data, which indicated that ambroxol showed higher cytotoxicity than calcium hydroxide paste [18]. In vitro research provides guidelines for in vivo studies, suggesting that ambroxol can be clinically used with acceptable cytotoxicity.

## 5 Conclusion

The null hypothesis was rejected. The pH of the association was more acidic. Solubility did not show significant differences over time. The combination did not negatively affect the cytotoxicity of calcium hydroxide, and its antimicrobial capacity was not impaired. Further research is necessary to consolidate this association as intracanal medication. A lower concentration of ambroxol may increase antimicrobial action without interfering with pH and solubility. Additionally, a different vehicle could reduce the cytotoxicity of this medication. Further investigation is still required.

## Author Contributions

All authors have contributed significantly to this project and article. Likewise, all authors are in agreement with this manuscript. Each person's contribution: Thaís Ferreira Rodrigues Mota: article writing, pH, solubility and antimicrobial analysis. Ana Flávia Balestrero Cassiano: cytotoxicity analysis. Pedro César Gomes Titato: antimicrobial analysis. Pedro Henrique Souza Calefi: pH and solubility analysis. Murilo Priori Alcalde: statistical analysis. Gisele Faria: cytotoxicity analysis, co‐supervision. Marco Antonio Hungaro Duarte: Academic Advisor, article correction.

## Funding

This work was supported by Fundação de Amparo à Pesquisa do Estado de São Paulo, 2021/05843‐2.

## Conflicts of Interest

The authors declare no conflicts of interest.

## Data Availability

The data that support the findings of this study are available from the corresponding author upon reasonable request.
